# Evolutionary rescue in a fluctuating environment: periodic versus quasi-periodic environmental changes

**DOI:** 10.1098/rspb.2023.0770

**Published:** 2023-05-31

**Authors:** Loïc Marrec, Claudia Bank

**Affiliations:** ^1^ Institut für Ökologie und Evolution, Universität Bern, Baltzerstrasse 6, 3012 Bern, Switzerland; ^2^ Swiss Institute of Bioinformatics, 1015 Lausanne, Switzerland

**Keywords:** evolutionary rescue, extinction, fluctuating environment

## Abstract

No environment is constant over time, and environmental fluctuations impact the outcome of evolutionary dynamics. Survival of a population not adapted to some environmental conditions is threatened unless, for example, a mutation rescues it, an eco-evolutionary process termed evolutionary rescue. We here investigate evolutionary rescue in an environment that fluctuates between a favourable state, in which the population grows, and a harsh state, in which the population declines. We develop a stochastic model that includes both population dynamics and genetics. We derive analytical predictions for the mean extinction time of a non-adapted population given that it is not rescued, the probability of rescue by a mutation, and the mean appearance time of a rescue mutant, which we validate using numerical simulations. We find that stochastic environmental fluctuations, resulting in quasi-periodic environmental changes, accelerate extinction and hinder evolutionary rescue compared with deterministic environmental fluctuations, resulting in periodic environmental changes. We demonstrate that high equilibrium population sizes and per capita growth rates maximize the chances of evolutionary rescue. We show that an imperfectly harsh environment, which does not fully prevent births but makes the death rate to birth rate ratio much greater than unity, has almost the same rescue probability as a perfectly harsh environment, which fully prevents births. Finally, we put our results in the context of antimicrobial resistance and conservation biology.

## 1. Introduction

Environmental change happens all around us and impacts the populations that experience it. For example, every living organism is exposed to climate change [1–4], and pathogenic microbes are exposed to varying drug concentrations [5,6], which threatens their survival. Populations too poorly adapted to changing environmental conditions may go extinct unless adaptive mutations counteract their decline, a process termed evolutionary rescue. Evolutionary rescue occurs through two different genetic routes: either the viable mutation rescuing the population pre-exists in the standing genetic variation or appears de novo [7]. Whether by mutations of standing genetic variation or de novo, an essential aim in theoretical biology is to predict whether evolutionary rescue will occur before extinction and which conditions favour adaptation [7–9].

Numerous theoretical works have shown that environmental fluctuations affect evolutionary dynamics. Specifically, analytical predictions were derived to assess the fate of a mutation in a fluctuating environment, which impacts either demography [10–12] or selection [12–15]. For example, these analytical predictions showed that a cyclic change in population size or selection coefficient (resembling a fluctuating environment) results in a mutant fixation probability that is also periodic as a function of the time of appearance. However, many of these models assume that environmental fluctuations do not impact demography and selection together, which is a simplification that overlooks a key aspect of evolutionary dynamics: the interaction of population dynamics and population genetics (but see [16,17] and appendix of [12]).

The interaction between population dynamics and genetics is all the more important as it exists everywhere in nature. For example, antimicrobial treatments are designed to decrease the size of microbial populations until their eradication. Treatment inhibits reproduction (and thus the appearance of mutations), but selects for antimicrobial-resistant mutants that may appear during drug therapy [5,6,18–24]. Similarly, climate change may cause extinction [25–27], but some animal species adapt quickly to stressful conditions and reverse their decline [28–30]. Importantly, the interaction between demography and selection can result in population decline, reducing genetic diversity, which could hinder evolutionary rescue [31]. To improve theoretical predictions and inference from experimental and empirical data, there is a need for mathematical models that make an explicit link between ecology, evolution and demography when quantifying the fate of a population evolving in a fluctuating environment [32,33]. One of the challenges to overcome is to go beyond the approximation that environmental and evolutionary time scales are decoupled [34,35]. Specifically, environmental effects are often self-averaged if environmental fluctuations are rapid [36], or a constant environment is assumed if environmental fluctuations are slow [37] (but see [11,14,38]). Another challenge is to derive exact analytical predictions that do not rely on deterministic or diffusion approximations, which have been shown to poorly describe extreme events such as extinction [39], a key factor for modelling evolutionary rescue.

In this paper, we develop a minimal model that integrates population dynamics and genetics to quantify evolutionary rescue in a fluctuating environment. Specifically, we study a haploid population evolving in an environment fluctuating between a favourable state, in which the population grows, and a harsh state, in which it declines. This fluctuating environment ultimately destines the population to extinction. However, if a mutation unaffected by environmental changes becomes fixed, the population is rescued from extinction. Importantly, we investigate the probability of evolutionary rescue using a stochastic framework with numerical and analytical tools, resulting in an exact computation of the population’s fate in a fluctuating environment. We quantify the impact of stochastic environmental fluctuations, resulting in quasi-periodic environmental changes, on evolutionary rescue compared with deterministic environmental fluctuations, resulting in periodic environmental changes. We also compare a perfectly harsh (i.e. fully birth-preventing) and an imperfectly harsh (i.e. not fully birth-preventing) environment and identify which growth parameters promote evolutionary rescue under different growth types.

## 2. Model and methods

### (a) A population model in a fluctuating environment

**Figure 1.**
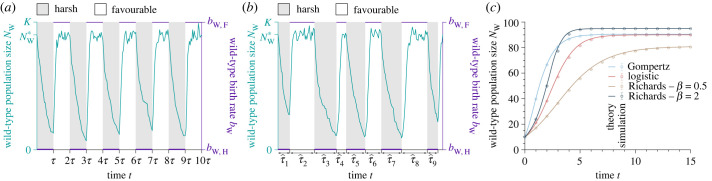
Illustration of the model—population dynamics in a fluctuating environment. Wild-type population size *N*_W_ and birth rate *b*_W_ versus time *t* with deterministic (*a*) and stochastic environmental fluctuations (*b*). In both panels, the solid line represents a realization of a simulation run under the logistic growth. The grey and white phases correspond to harsh and favourable environments, respectively. (*c*) Wild-type population size *N*_W_ versus time *t* for different population growth types in a constant favourable environment. Solid lines represent analytical predictions, and data points show simulated data averaged over 10^4^ stochastic realizations. Error bars correspond to the 95% confidence intervals. Parameter values: wild-type birth rate in a favourable environment *b*_W,F_ = 1, wild-type birth rate in a harsh environment *b*_W,H_ = 0, wild-type death rate *d*_W_ = 0.1, carrying capacity *K* = 100, and equilibrium wild-type population size $N_{W}^{*}=90$.

We study a wild-type population of size *N*_W_, which can vary over time and remains lower than a constant carrying capacity *K*. Each wild-type individual has the same birth rate *b*_W,*α*_, which depends on the environmental state *α*, and death rate *d*_W_ independent of the environment. The population follows a logistic growth in which the per capita birth rate satisfies *b*_W,*α*_(1 − *N*_W_/*K*), and the per capita death rate is equal to the intrinsic death rate. Note that the per capita birth rate is always positive since the population size does not exceed the carrying capacity. We also present results for the Gompertz and Richards (also called theta-logistic [40]) growths, whose per capita birth rates satisfy *b*_W,*α*_log(*K*/*N*_W_) and $b_{W,\alpha }(1-{\left(N_{W}/K\right)}^{\beta })$, respectively (figure 1*c*) [41]. These growth types, which are used to fit population growth data [42], have different equilibrium sizes for a given parameter value set (i.e. carrying capacity, birth rate and death rate). Specifically, in the case where *b*_W,*α*_ > *d*_W_, the equilibrium population size $N_{W}^{*}$ is equal to *K*(1 − *d*_W_/*b*_W,*α*_), *K*(1 − *d*_W_/*b*_W,*α*_)^1/*β*^, and $Ke^{-d_{W}/b_{W,\alpha }}$ for the logistic, Richards and Gompertz models, respectively. Consequently, the growth types have different per capita growth rates as they assume different density dependence of the birth rate. These differences affect the probability of evolutionary rescue. The population evolves in an environment that fluctuates between two states, namely favourable F and harsh H, which impacts only the birth rate. In a favourable environment, the intrinsic birth rate is larger than the intrinsic death rate (i.e. *b*_W,F_ > *d*_W_) so that the population grows towards its equilibrium size $N_{W}^{*}$. Conversely, in a harsh environment, the intrinsic birth rate is lower than the intrinsic death rate (i.e. *b*_W,H_ < *d*_W_), so that the population declines towards extinction. An example of a simulation run is shown in figure 1*a*. Each environment, whether harsh or favourable, lasts for a duration *τ*, sampled from the probability density function $F_{\tau }$. In the case of deterministic fluctuations, resulting in periodic environmental changes, we set $F_{\tau }(t)=\delta (t-\tau )$, in which *δ* is the Dirac delta. In the case of stochastic fluctuations, resulting in quasi-periodic environmental changes, the environment duration is drawn from a biased normal distribution of mean *τ* and standard deviation *σ* given by $F_{\tau }(t)=\frac{2e^{-\frac{1}{2}{\left(\frac{t-\tau }{\sigma }\right)}^{2}}}{\sigma \sqrt{2\pi }(1+\mathrm{Erf}(t/(\sigma \sqrt{2\pi })))}$ that excludes negative values (figure 1*b*).

A generalist mutant appears upon reproduction with probability *μ* and has a birth rate *b*_M_ and a death rate *d*_M_ that are both constant across environments. We assume that the population is initially composed of wild-type individuals whose number is equal to the equilibrium population size $N_{W}^{*}$, which results from the demographic balance obtained when births and deaths offset each other. As a reminder, for the logistic growth, the equilibrium population size is equal to *K*(1 − *d*_W_/*b*_W,F_). In addition to de novo mutant appearances, a mutant may pre-exist before the population begins to experience environmental fluctuations. We then consider the two cases: with and without pre-existing mutants.

Our analytical approach uses methods from birth–death processes described by master equations [43,44]. Our simulations are based on a Gillespie algorithm and incorporate individual stochastic division, mutation and death events [45,46]. The algorithm we used is detailed in the electronic supplementary material.

We present a few extensions to our model in the electronic supplementary material. Among these extensions, we consider the case of a mutant sensitive to environmental fluctuations, i.e. whose birth rate depends on the environment. We also consider the case of three environments, one of which is intermediate between the harsh and favourable environments and in which the wild-type birth rate is not fully restored. These extensions intend to show the robustness of our model and analytical predictions.

### (b) Time scales of environmental fluctuations

In the fluctuating environment, either the population goes extinct at time *T*_0_, or a mutant rescues the population before *T*_0_. The evolutionary outcome crucially depends on how the environmental time scale *τ* compares to the population’s lifetime *τ*_0,H_ in a harsh environment. In the limit of large *τ*, for *τ* ≫ *τ*_0,H_, very slow environmental fluctuations lead to rapid extinction (i.e. *T*_0_ = *τ*_0,H_) because the harsh environment lasts much longer than the population lifetime in a harsh environment (see electronic supplementary material, figure S1a). Here, the rapid extinction leaves little (if *b*_W,H_ > 0 and *d*_W_/*b*_W,H_ ≫ 1) or no opportunity (if *b*_W,H_ = 0) for rescue mutants to appear and therefore the rescue probability by de novo mutations *p*_r,DN_ is likely to be zero. In the limit of small *τ*, for *τ* ≪ *τ*_0,H_, very rapid environmental fluctuations make the population persist long enough for mutations to arise and rescue it almost surely. In the particular case of very fast environmental fluctuations, the evolutionary dynamics can be described by a constant environment with an averaged birth rate ${\tilde{b}}_{W}=(b_{W,F}+b_{W,H})/2$ and an effective equilibrium size ${\tilde{N}}_{W}^{*}$ satisfying $0<{\tilde{N}}_{W}^{*}<N_{W}^{*}$ (see electronic supplementary material, figure S1b). Note that, although rapid environmental fluctuations maintain the population in an equilibrium state, its extinction time ${\tilde{T}}_{0}$ is reduced compared with if it remained indefinitely in a favourable environment. In the case of an effective constant environment, the mean appearance time of a beneficial mutant of selection coefficient $\tilde{s}$ (i.e. $\tilde{s}=b_{M}d_{W}/({\tilde{b}}_{W}d_{M})-1>0$) that becomes fixed is given by ${\tilde{\tau }}_{\mathrm{af}}=1/(\mu \;{\tilde{N}}_{W}^{*}\;d_{W}\tilde{s})$ (see electronic supplementary material, figure S2 and text, for derivation). If this time is much shorter than the mean extinction time ${\tilde{T}}_{0}$, the rescue probability by de novo mutations *p*_r,DN_ is likely to be one.

Whether a mutant pre-exists before the population starts experiencing environmental fluctuations does not impact the key time scales that determine if evolutionary rescue is likely (i.e. environmental time scale *τ* and the lifetime of the population *τ*_0,H_). However, the pre-existence of a mutant changes the lower bound of the rescue probability from 0 to *p*_r,SGV_, where *p*_r,SGV_ is the rescue probability due to standing genetic variation. A pre-existent mutant may fix during the first harsh environment, thus increasing the total rescue probability compared to only de novo mutations.

In the following, we focus on non-trivial cases in which the environmental time scale is of the same order of magnitude as the population lifetime in a harsh environment (i.e. *τ* ∼ *τ*_0,H_). In such non-trivial cases, the total rescue probability *p*_r_ is likely to satisfy 0 < *p*_r_ < 1 for de novo mutations, or *p*_r,SGV_ < *p*_r_ < 1 with de novo mutations and pre-existent mutants.

### (c) Stochastic dynamics of the wild-type population and appearance and fixation of a mutant

For reasons of article length, derivations of analytical predictions giving the dynamics of wild-type population size as a function of time in a fluctuating environment, the probabilities of wild-type population extinction in each environmental state in the absence of evolutionary rescue, and the probability of mutant appearance and fixation are detailed in the electronic supplementary material.

## 3. Heuristic analysis

### (a) Two different extinction mechanisms contribute to failed evolutionary rescue

Environmental fluctuations decrease the persistence time of a population if they induce new paths to extinction. In a harsh environment, the population declines because the death rate exceeds the birth rate. If a harsh environment duration *τ* is longer than the survival time *τ*_0,H_, the population goes extinct. The survival time in a harsh environment is stochastic and extinction occurs with probability $p_{0,H}=\int_{0}^{\infty }F_{\tau }(t)P_{H}(0,t|N_{W}^{*})dt$ (see electronic supplementary material, equation (S2)).

A second path to extinction exists in a favourable environment. If the population survives the previous harsh environment, it possibly starts the new favourable environment with few individuals. Small initial population sizes lead to strong demographic noise that may drive the population to extinction with probability $p_{0,F}=\sum_{N_{W}=1}^{N_{W}^{*}}$ $\left(\int_{0}^{\infty }F_{\tau }(t)P_{H}(N_{W},t|N_{W}^{*})dt)P_{F}^{\infty }(0|N_{W})/(\int_{0}^{\infty }F_{\tau }(t)P_{H}(0,t|N_{W}^{*})dt\right)$ [39]. An example of each extinction mechanism is shown in the electronic supplementary material, figure S5. The proportion of extinctions occurring in a favourable environment, which is given by *ω*_F_ ≈ *p*_0,F_/(*p*_0,F_ + *p*_0,H_), is expected to decrease with increasing environment duration. The longer a harsh environment, the more certain it drives the population to extinction. Conversely, short harsh environments do not drive the population to extinction but, in some cases, decrease the population size enough to lead to rapid extinction in the next favourable environment.

### (b) Stochastic environmental fluctuations can increase or decrease the rescue probability compared to deterministic environmental fluctuations

As explained before, the fate of a population depends on how the survival time of the wild-type in a harsh environment compares to the environment duration. If the environmental fluctuations are deterministic and the mean environment duration is shorter than the mean survival time in a harsh environment (i.e. *τ* < *τ*_0,H_), a harsh environment tends to be too short to drive the population to extinction. However, if the environmental fluctuations are stochastic, some harsh environments are longer than average, which favours extinction and decreases the total extinction time and the total rescue probability. Although the extinction time does not impact the fixation probability of potential pre-existing mutants resulting from standing genetic variation, its decrease induces a decrease in the probability of mutant appearance and, thus, in the probability of rescue by de novo mutations. If the environmental fluctuations are deterministic and the mean environment duration is longer than the mean survival time in a harsh environment (i.e. *τ*_0,H_ < *τ*), a harsh environment tends to be long enough to drive the population to extinction. However, if the environmental fluctuations are stochastic, some harsh environments are shorter than average, which favours population survival and increases the total extinction time and the total rescue probability as more mutants can appear and potentially become fixed. In summary, no matter whether the environmental fluctuations are deterministic or stochastic, the total extinction time and the probability of rescue decrease as the environment duration increases. However, for a mean environment duration shorter than the mean survival time in a harsh environment (i.e. *τ* < *τ*_0,H_), the stronger the environmental stochasticity (i.e. the larger the standard deviation *σ*, which results in a more irregular periodicity of environmental changes), the lower the total extinction time and the rescue probability. The opposite is valid for a mean environment duration longer than the mean survival time in a harsh environment (i.e. *τ*_0,H_ < *τ*).

### (c) Low birth rates in a harsh environment leave rescue probabilities almost unchanged compared with zero birth rates

A harsh environment induces a wild-type birth rate lower than the death rate. Specifically, a perfectly harsh environment fully prevents births, whereas an imperfectly harsh environment allows for a small number of births. As long as the birth rate in a harsh environment is much lower than the death rate (i.e. *d*_W_/*b*_W,H_ ≫ 1; e.g. *d*_W_/*b*_W,H_ = 10), the population is driven to extinction on a time scale equal to $\tau_{0,H}\approx \log(N_{W}^{*})/d_{W}$ (see electronic supplementary material, figure S6). Thus, the mean total extinction time should not significantly differ between perfectly and imperfectly harsh environments that satisfy *d*_W_/*b*_W,H_ ≫ 1. However, *b*_W,H_ may impact the rescue probability by de novo mutations as it determines how many births occur and how many mutants appear. Specifically, there are $N_{\mathrm{birth}}\approx b_{W,H}N_{W}^{*}(2K-N_{W}^{*})/(2d_{W}K)$ births in each harsh environment and the probability that at least one mutant appears is given by $1-{\left(1-\mu \right)}^{N_{\mathrm{birth}}}$ (see electronic supplementary material, figure S7). Thus, the larger the birth rate *b*_W,H_ and the mutation probability *μ*, the more mutants appear in a harsh environment. However, if the death rate to birth rate ratio satisfies *d*_W_/*b*_W,H_ ≫ 1, the number of births in a harsh environment is expected to be very small, and the number of mutants that appear is much smaller. If a mutant from standing genetic variation exists before the population starts experiencing environmental fluctuations, whether the environment is perfectly or imperfectly harsh should not change its fixation probability as long as the wild-type population declines in the first harsh environment. Therefore, the total rescue probability with an imperfectly harsh environment is likely similar to that with a perfectly harsh environment.

### (d) Rescue probability depends on population growth types

In addition to studying evolutionary rescue under the logistic growth, we also present results for the Gompertz and Richards growths. For a given parameter value set (i.e. carrying capacity, birth rate and death rate), each of these growth types has a different equilibrium size $N_{W}^{*}$ and growth rate, which affect the total extinction time and the rescue probability. First, the larger the equilibrium size, the longer it takes for the population to go extinct in a harsh environment since $\tau_{0,H}\approx \log(N_{W}^{*})/d_{W}$. Thus, the probability of extinction in a harsh environment *p*_0,H_ at a given environment duration *τ* decreases as the equilibrium size increases. Second, the faster the growth, the lower the demographic stochasticity. The probability of rapid extinction *p*_0,F_ of a population whose initial size *N*_W,0_ is very small compared with its equilibrium size is given by ${\left(d_{W}/b_{W,F}\right)}^{N_{W,0}}$ for the logistic and Richards growths and ${\left(d_{W}/(b_{W,F}\log(K))\right)}^{N_{W,0}}$ for the Gompertz growth. Thus, the probability of extinction *p*_0,F_ for a given environment duration decreases for populations with a higher growth rate. Naturally, the lower the probabilities of extinction in harsh and favourable environments, the longer the total extinction time. An increased total extinction time leaves more opportunities for mutants to appear and become fixed. Moreover, a larger growth rate results in more births, resulting in more mutants and, therefore, a higher rescue probability. As shown in figure 1*c*), the Gompertz model and the Richards model for *β* = 2 have the largest equilibrium population sizes and growth rates before the saturation phase. As a result, these population growth models are likely to favour evolutionary rescue compared with the logistic model and the Richards model for *β* = 0.5. As a reminder, *β* > 1 implies a faster-than-linear decrease in the wild-type per capita birth rate as the population density *N*/*K* increases for the Richards model. Conversely, *β* < 1 implies a slower-than-linear decrease in the wild-type per capita birth rate as the population density *N*/*K* increases. We present in the electronic supplementary material another comparison based on setting an equal equilibrium size by adjusting either the carrying capacity or the birth rate.

## 4. Formal analysis

### (a) Extinction time

From the extinction probabilities, namely *p*_0,F_ and *p*_0,H_ (see Model and methods and Heuristic analysis), we compute the probability $P_{q_{F}}$ that the population undergoes *q*_F_ favourable environments before it goes extinct as

4.1 $$P_{q_{F}}(k)={\left(1-p_{0,H}\right)}^{k}{\left(1-p_{0,F}\right)}^{k}(p_{0,H}+(1-p_{0,H})p_{0,F}).$$

The favourable environments in which a rapid extinction occurs are excluded from this count because we focus on the favourable environments in which a mutant is most likely to appear. We obtain the mean number of favourable environments before extinction by calculating $\left\langle q_{F}\rangle =\sum_{k=0}^{\infty }kP_{q_{F}}(k\right)$, which yields

4.2 $$\langle q_{F}\rangle =\frac{-1+p_{0,H}+p_{0,F}-p_{0,H}p_{0,F}}{-p_{0,H}-p_{0,F}+p_{0,H}p_{0,F}}.$$

Equation (4.2) shows that both extinction mechanisms (extinction in a harsh environment versus extinction due to low numbers at the beginning of a favourable environment) are important in assessing population persistence. The probabilities *p*_0,F_ and *p*_0,H_ increase as the environment duration increases (see electronic supplementary material, figure S8a,b), reducing 〈*q*_F_〉. Specifically, the probability of extinction in a harsh environment ranges from 0 to 1 (i.e. 0 < *p*_0,H_ < 1) since short harsh environments do not leave enough time for the population to go extinct. By contrast, long harsh environments surely drive it to extinction before the next environmental change. The probability of extinction in a favourable environment ranges from 0 to *d*_W_/*b*_W,H_ (i.e. 0 < *p*_0,F_ < *d*_W_/*b*_W,H_), where *d*_W_/*b*_W,H_ is equal to the probability that a population starting with one individual rapidly goes to extinction. Using equation (4.2) and the proportion *ω*_F_ of extinction in a favourable environment (i.e. *ω*_F_ ≈ *p*_0,F_/(*p*_0,F_ + *p*_0,H_)), we derive the mean total extinction time as

4.3 $$T_{0}=2\langle q_{F}\rangle \tau +(1-\omega_{F})\tau_{0,H}+\omega_{F}\tau_{0,F}.$$

**Figure 2.**
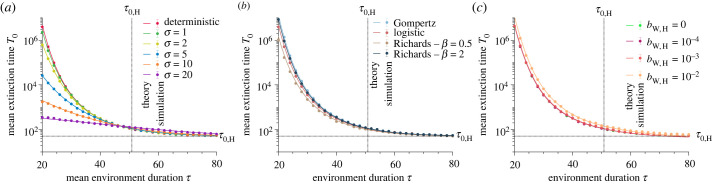
Extinction time decreases as environment duration increases. Mean extinction *T*_0_ time versus environment duration *τ*. Panel (*a*) compares deterministic and stochastic environmental fluctuations for the logistic model, panel (*b*) compares different population growth models, and panel (*c*) compares perfectly and imperfectly harsh environments for the logistic model. The solid lines represent analytical predictions, and the points simulated data averaged over 10^4^ stochastic realizations. The error bars correspond to the 95% confidence intervals but are smaller than the markers. Vertical dotted lines represent the mean survival time in a harsh environment *τ*_0,H_. Parameter values: wild-type birth rate in a favourable environment *b*_W,F_ = 1, wild-type birth rate in a harsh environment *b*_W,H_ = 0 (in *a* and *b*), wild-type death rate *d*_W_ = 0.1, carrying capacity *K* = 100, and equilibrium wild-type population size $N_{W}^{*}=90$.

Independent of whether extinction occurs in a favourable or harsh environment, the population persists during 〈*q*_F_〉 epochs of a mean duration *τ* plus the mean survival time in a favourable (respectively, harsh) environment, given that the population goes extinct, weighted by the probability that extinction occurs in a favourable (respectively, harsh) environment. The mean total extinction time ranges from *τ*_0,H_ to ${\tilde{T}}_{0}$ (i.e. $\tau_{0,H}\leq T_{0}\leq {\tilde{T}}_{0}$), where the mean survival time *τ*_0,H_ in a harsh environment is obtained for very long environment durations. By contrast, the mean extinction time ${\tilde{T}}_{0}$ in an effectively constant environment is obtained for very short environment durations. The proportion *ω*_F_ of extinction in a favourable environment decreases as the environment duration increases (see electronic supplementary material, figure S8c), reducing *T*_0_. If the ratio of death rate to birth rate in a favourable environment is much smaller than unity (i.e. *d*_W_ ≪ *b*_W,F_), we can assume that rapid extinction in a favourable environment occurs if the population starts with only a single individual, hence *τ*_0,F_ ≈ 1/*d*_W_. The extinction time *τ*_0,H_ in a harsh environment is then approximately equal to $\log(N_{W}^{*})/d_{W}$ if *τ* > *τ*_0,H_, or to *τ* otherwise. Our analytical predictions accurately predict the simulated data (see figure 2; see also electronic supplementary material, figure S9 for 〈*q*_F_〉). As reported in figure 2*a*, the greater the environmental stochasticity (i.e. the larger the standard deviation *σ*), the smaller the mean total extinction time *T*_0_. Even small values of the standard deviation of environmental stochasticity *σ* dramatically affect population persistence. The mean total extinction times for deterministic and stochastic fluctuations intersect around the mean survival time in a harsh environment. Beyond this time, the population persists longer in an environment with highly stochastic fluctuations, but the difference to the result for deterministic fluctuations becomes much smaller. As reported in figure 2*b*, populations growing under a growth type with a larger equilibrium size and growth rate have an increased extinction time. This difference fades as the environment duration increases since extinction occurs mainly in a harsh environment, where the extinction probability is independent of the growth type. Electronic supplementary material, figure S15b shows that the mean extinction time is similar for every population growth model with the same equilibrium size, wild-type birth rate and wild-type death rate, but different carrying capacities. Finally, figure 2*c* shows that for any ratio *d*_W_/*b*_W,H_ much greater than unity, the mean total extinction time is equal because the probability of extinction in a harsh environment is the same as if *b*_W,H_ = 0. Note that the maximum population size scales the window of environment durations that lead to non-trivial rescue probabilities (i.e. *τ* ∼ *τ*_0,H_ so that 0 < *p*_r_ < 1). Specifically, the mean survival time in a harsh environment is given by $\tau_{0,H}\approx \log(N_{W}^{*})/d_{W}$. By contrast, its variance is approximately equal to $1/d_{W}^{2}$ (both quantities can be derived from equation (S2) in the electronic supplementary material). We present additional results for different maximum population sizes as a function of *τ*/*τ*_0,H_ in electronic supplementary material, figure S10.

### (b) Probability of rescue by de novo mutations

Using the mean number 〈*q*_F_〉 of favourable environments that the population undergoes, we calculate the probability that a generalist mutant (i.e. one not affected by environmental fluctuations) appears and takes over the population before extinction occurs given that no mutant did it before. We obtain

4.4 $$p_{r,\mathrm{DN}}=\int_{0}^{\infty }F_{T_{0}}(t)p_{\mathrm{af}}(t)\;dt,$$

where $F_{T_{0}}$ is the probability density function of the total extinction time. Equation (4.4) is simplified by separating the contribution of the favourable and harsh environments. Either the mutant appears in a favourable environment while the population is growing or in a harsh environment if the division is not fully hindered. Thus, the rescue probability by de novo mutations *p*_r,DN_ reads

4.5 $$p_{r,\mathrm{DN}}=\sum_{k=0}^{\infty }P_{q_{F}}(k)(1-e^{-(k+1)\Sigma_{H}-k\Sigma_{F}}),$$

where

4.6 $$\Sigma_{H}=\mu b_{W,H}\int_{0}^{\tau }{\left\langle N_{W}\right\rangle }_{H}(t)\left(1-\frac{{\left\langle N_{W}\right\rangle }_{H}(t)}{K}\right)p_{\mathrm{fix}}(t)\;dt$$

and

4.7 $$\Sigma_{F}=\mu b_{W,F}\int_{0}^{\tau }{\left\langle N_{W}\right\rangle }_{F}(t)\left(1-\frac{{\left\langle N_{W}\right\rangle }_{F}(t)}{K}\right)p_{\mathrm{fix}}(t+\tau )\;dt.$$

**Figure 3.**
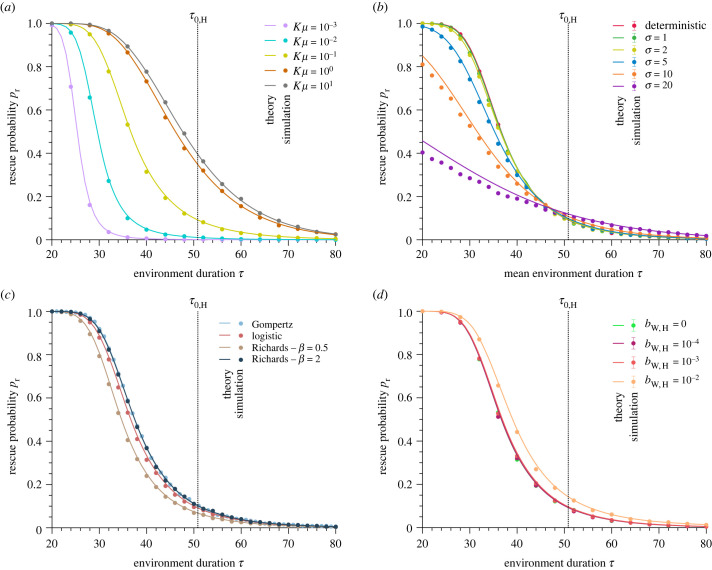
Rescue probability decreases as environment duration increases. Rescue probability *p*_r_ versus environment duration *τ* assuming no pre-existent mutants (i.e. *p*_r_ = *p*_r,DN_; only de novo mutations). Panel (*a*) compares different mutational influxes *Kμ*, panel (*b*) deterministic and stochastic environmental fluctuations for the logistic growth, panel (*c*) different population growth types, and panel (*d*) perfectly and imperfectly harsh environments for the logistic growth. The solid lines represent analytical predictions, and the points simulated data averaged over 10^4^ stochastic realizations. Vertical dotted lines represent the mean survival time in a harsh environment *τ*_0,H_. Parameter values: wild-type birth rate in a favourable environment *b*_W,F_ = 1, wild-type birth rate in a harsh environment *b*_W,H_ = 0 (in *a*, *b* and *c*), wild-type death rate *d*_W_ = 0.1, mutant birth rate *b*_M_ = 1, mutant death rate *d*_M_ = 0.1, carrying capacity *K* = 100, mutation probability upon division *μ* = 10^−3^ (in b, c and d) and equilibrium wild-type population size $N_{W}^{*}=90$.

The quantity *μb*_W_(*t*)(1 − 〈*N*_W_〉(*t*)/*K*)〈*N*_W_〉(*t*) is the mean number of mutants that appear between *t* and *t* + d*t*, to which we applied the mean-field approximation 〈(1 − *N*_W_(*t*)/*K*)*N*_W_(*t*)〉 ≈ (1 − 〈*N*_W_〉(*t*)/*K*)〈*N*_W_〉(*t*). Our analytical predictions match the simulated data very well (figure 3). In particular, figure 3*a* shows that equation (4.5) is valid from the rare to the frequent mutation regime. All panels highlight the transition from the regime of fast fluctuations, in which *p*_r,DN_ ≈ 1, to slow fluctuations, in which *p*_r,DN_ ≈ 0. This transition is more abrupt for rare mutations than for frequent mutations. The more mutants there are, the more likely one mutant becomes fixed and rescues the population before extinction, hence the higher probability of rescue by de novo mutations at a given environment duration. As reported in figure 3*b*, environmental stochasticity decreases the chances of evolutionary rescue because it also decreases the mean total extinction time. Population growth types with the highest growth rates and equilibrium sizes have the highest probabilities of rescue by de novo mutations at a given environment duration because they lead to more mutant appearances per unit of time (see figure 3*c*; electronic supplementary material, figure S15c). As shown in figure 3*d*, a harsh environment that does not fully prevent the reproduction of individuals leaves more opportunities for mutants to appear, resulting in a higher rescue probability.

The probability of rescue by de novo mutations is independent of the carrying capacity *K* at a given normalized environment duration *τ*/*τ*_0,H_ if the mutational influx *Kμ* is constant (see electronic supplementary material, figure S11). The carrying capacity determines the environment duration window in which the rescue probability transitions from 1 to 0 through the mean survival time in a harsh environment. The product *Kμ* determines the number of mutants that appear per unit of time.

### (c) Total probability of rescue

Now that we have determined the probability of rescue by de novo mutations, let us hypothesize that a single mutant from standing genetic variation pre-exists when the environment starts fluctuating. This pre-existing mutant represents an additional opportunity to rescue the population from extinction. More specifically, this mutant fixes with probability *p*_r,SGV_ = *p*_fix_(0) (see electronic supplementary material, equation (S7)), and thus the total probability of evolutionary rescue, i.e. combining standing genetic variation and de novo mutations, is given by [31]

4.8 $$p_{r}=p_{r,\mathrm{SGV}}+(1-p_{r,\mathrm{SGV}})p_{r,\mathrm{DN}},$$

where *p*_r,DN_ satisfies equation (4.5). As shown in electronic supplementary material, figure S17, there is a good agreement between the simulated data and our analytical predictions. Unlike the case with only de novo mutations, where the probability of rescue ranges from 0 to 1, the case with the additional standing genetic variation ranges from *p*_r,SGV_ to 1. Thus, the comparison between figure 3 and electronic supplementary material, figure S17 allows us to identify each genetic route’s contribution to the evolutionary rescue of the population. Specifically, de novo mutations contribute to population rescue only at environment durations shorter than the mean survival time of the population in a harsh environment. Conversely, the contribution of standing genetic variation does not depend on the environment duration. It, therefore, allows rescuing the population from extinction for environment durations longer than the mean survival time of the population in a harsh environment.

### (d) Appearance time of a de novo mutant rescuing the population

We derive the mean appearance time *τ*_af_ of a de novo mutant that fixes, given that the population is rescued by a de novo mutation, as

4.9 $$\tau_{\mathrm{af}}=(2\langle q_{\mathrm{af},F}\rangle -1)\tau +t_{\mathrm{af}}.$$

The mean number 〈*q*_af,F_〉 of favourable environments that occur before a mutant appears and fixes, given that the population is rescued, is given by

**Figure 4.**
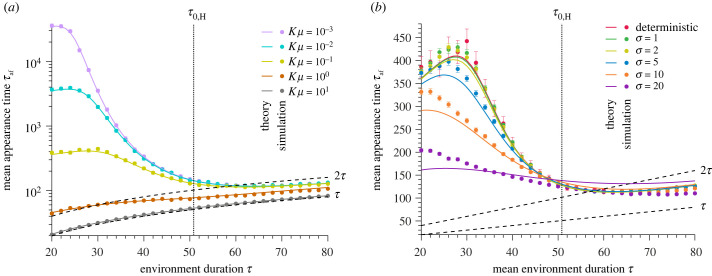
The larger the mutation probability upon division and the longer the environment duration, the earlier the rescue mutant appears. Mean appearance time of a mutant that rescues the population *τ*_af_ versus environment duration *τ* given that the population is rescued by a de novo mutation. Panel (*a*) compares different mutational influxes *Kμ* and panel (*b*) deterministic and stochastic environmental fluctuations for the logistic growth. The solid lines represent analytical predictions, and the points simulated data averaged over 10^4^ stochastic realizations. The error bars correspond to the 95% confidence intervals. Vertical dotted lines represent the mean survival time in a harsh environment *τ*_0,H_. Parameter values: wild-type birth rate in a favourable environment *b*_W,F_ = 1, wild-type birth rate in a harsh environment *b*_W,H_ = 0, wild-type death rate *d*_W_ = 0.1, mutant birth rate *b*_M_ = 1, mutant death rate *d*_M_ = 0.1, carrying capacity *K* = 100, mutation probability upon division *μ* = 10^−3^ (in *b*), and equilibrium wild-type population size $N_{W}^{*}=90$.

4.10 $$\langle q_{\mathrm{af},F}\rangle =\sum_{k=0}^{+\infty }P_{q_{F}}(k)\sum_{q=0}^{k}qe^{-(q-1)\Sigma_{F}}(1-e^{-\Sigma_{F}})/p_{r},$$

and *t*_af_ is the mean appearance time of a mutant that becomes fixed in a favourable environment. Since the mean total extinction time and rescue probability are similar for *d*_W_/*b*_W,H_ ≫ 1 (figures 2 and 3), we assume that a mutant is unlikely to emerge in a harsh environment. In the moderate to frequent mutation regime and regardless of environment duration, the mutant that rescues the population appears during the first favourable environment (figures 4; electronic supplementary material, figure S12). Then *τ*_af_ increases as *τ* increases. If mutations are rare, the number of favourable environments before a rescue mutant appears decreases as the environment duration increases. More precisely, 〈*q*_af,F_〉 converges to unity when the environment duration is longer than the survival time in a harsh environment. The population goes extinct quickly for such an environment duration, so the mutant must appear in the first favourable environments. Our results confirm previous observations that the mutant rescuing the population from extinction tends to appear just before an environmental change from a favourable to a harsh environment [17,49,50].

## 5. Discussion

Whether it is microbes subjected to varying antimicrobial concentrations or animal species caught up in climate change, populations experience environmental changes threatening their survival. Determining whether populations adapt or perish is fundamental in many fields, from antimicrobial resistance to conservation biology. In this paper, we develop a minimal model to address evolutionary rescue in a fluctuating environment. We consider deterministic and stochastic fluctuations resulting in periodic and quasi-periodic environmental changes. We fully analyse our model using analytical and numerical tools from stochastic processes. Specifically, we derive equations for the extinction time, the rescue probability, and the appearance time of a rescue mutant and validate them with numerical simulations. Our approach predicts the evolutionary rescue of a population in a fluctuating environment causing simultaneous demography and selection changes. In particular, our analytical approach extends the results of Uecker & Hermisson [12] and Marrec & Bitbol [17] to a case where the extinction of the population is not deterministically determined (e.g. exponential population decay to extinction) and, thus, requires the calculation of the extinction time.

### (a) Stochastic environmental fluctuations accelerate extinction and hinder evolutionary rescue compared to deterministic fluctuations

Our study quantifies the probability of evolutionary rescue of a population evolving in an environment that fluctuates, either deterministically or stochastically, between a harsh state (i.e. causing a population decline) and a favourable state (i.e. allowing population growth). These deterministic and stochastic environmental fluctuations result in periodic and quasi-periodic environmental changes, respectively (figure 1*a*,*b*). We show that environmental and survival time scales determine whether stochastic environmental fluctuations favour evolutionary rescue compared to deterministic ones. Specifically, we prove that stochastic environmental fluctuations with a mean environment duration shorter than the survival time in a harsh environment dramatically decrease the mean total extinction time, the rescue probability, and the mean appearance time of a rescue mutant (figures 2*a*, 3*b* and 4*b*). When the mean environment duration is shorter than the mean survival time, environmental stochasticity leads to longer-than-average duration, thus facilitating extinction. Conversely, stochastic environmental fluctuations with a mean environment duration that is longer than the mean survival time of the population in a harsh environment very slightly increase the mean total extinction time and the rescue probability but do not significantly affect the mean appearance time of a rescue mutant.

Stochasticity plays a crucial role in evolution at different scales, from mutation at the gene level to environment at the population level [51]. Temporal changes in the environment of populations can be seasonal, fairly predictable or noisy [52,53]. The seasons are a concrete example of periodic changes, whereas temperature variation is an excellent example of environmental noise. In both cases, environmental changes induce variations in natural selection over time, which explains why population adaptation in a fluctuating environment has been the subject of numerous works. Similar to ours, some investigated the impact of a periodically changing environment and identified parameter regimes where evolutionary rescue overrides extinction [49,50,54,55]. Such environmental change patterns are specifically relevant for modelling drug treatment. It is worth noting that environmental fluctuations can be more continuous in the wild, which is why other studies have focused on autocorrelated environments [56–59]. For example, an experimental work in which microalgae were subjected to a fluctuating salinity showed that the fluctuation colour (i.e. the temporal autocorrelation of the salinity fluctuations) strongly impacted the population dynamics and extinction rates of the microalgae [60,61].

Relating our results to a public health perspective, our model may represent a treatment with a biostatic drug, which inhibits microbial division. Our model does not explicitly account for pharmacodynamics (i.e. the drug’s biochemical and physiological effect) or pharmacokinetics (i.e. drug degradation over time) inherent to any drug during treatment. However, the pharmacodynamic curve (i.e. net growth rate versus drug concentration) is often very steep around the minimum inhibition concentration (MIC) [18], which could justify our binary approximation (i.e. harsh and favourable environments). Here, the microbial division is impaired when the drug concentration is above the MIC (harsh environment). By contrast, the microbial division is not impacted when the drug concentration drops below the MIC (favourable environment). Moreover, as shown in the electronic supplementary material (see electronic supplementary material, figure S14), our model is easily extendable to the case of an antimicrobial-resistant mutant carrying a resistance cost (i.e. having a lower division rate than the wild-type without antimicrobials) [62] and being slightly sensitive to antimicrobials without being led to extinction [63]. Under this scenario, we evaluate the risk of antimicrobial resistance evolution during therapy and, thus, treatment failure [9,64]. Similar to a previous theoretical work [17], we show that fluctuations in antimicrobial concentration play a role in resistance evolution. For example, we find that rapid deterministic fluctuations favour the evolution of resistance over a constant environment. We extend [17] by showing that rapid stochastic fluctuations of the drug concentration can decrease the risk of resistance evolution. Furthermore, our analytical prediction for the probability of evolutionary rescue is valid across regimes of fast to slow environmental fluctuations. Our analytical prediction complements the work of [17], whose analytical results have been derived in the limit of fast or slow fluctuation regimes. Specifically, whereas the analytical results of [17] rely on the approximation that a single environmental change occurs before the extinction or rescue of the population for fast environmental fluctuations or an effectively constant environment for slow environmental fluctuations, the validity of our approach across regimes relies on counting the number of favourable environments until extinction in the absence of evolutionary rescue.

Long-term therapies involving multiple dosing are subject to imperfect adherence to treatment, i.e. patients often fail to follow the exact treatment plan [65–67]. With this in mind, the impact of missed doses on resistance evolution was theoretically investigated in [21], which showed that non-adherence allows resistant strains to grow. In our model, stochastic fluctuations may result from another form of imperfect adherence: doses taken at irregular intervals. These doses taken at irregular intervals lead the serum drug concentration to go above and below the MIC irregularly, resulting in a quasi-periodic drug action. Surprisingly, our model suggests that a biostatic antimicrobial treatment, taken at irregular intervals, may hinder resistance evolution rather than accelerate it.

In summary, our theoretical work can inform the design of drug treatments that prevent the evolution of resistance by choosing the best type of antimicrobial and the time interval between each dose. A possible extension would be to compare two types of antimicrobial, namely biostatic (i.e. hindering microbial division) and biocidal (i.e. killing microbes) [18,19], by including environment-dependent death rates. We expect that biocidal drugs accelerate extinction compared to biostatic drugs while at the same time promoting evolutionary rescue. That is because since biocidal drugs do not prevent cell division, more mutants appear, which increases the probability of evolutionary rescue.

### (b) High equilibrium population sizes and growth rates slow down extinction and favour evolutionary rescue

Our model includes an explicit link between ecology, evolution and demography: environmental fluctuations impact the wild-type birth rate, affecting the population size and the selective advantage of the mutant. Thus, our work does not rely on the common assumption that ecology and evolution are uncoupled when studying the genetics of adaptation [68]. This assumption was already relieved in theoretical studies that have analytically predicted adaptation in a fluctuating environment inducing changes in either population size or selection coefficient, but not both together [12,14,38]. The analysis of our eco-evolutionary model shows that the underlying growth type (i.e. the underlying growth model; figure 1*c*) plays an essential role in the population’s fate. Specifically, we show that growth types with larger equilibrium sizes lengthen the mean survival time of the population in a harsh environment, and growth types with higher growth rates decrease the probability of rapid extinction in a favourable environment. As a result, large equilibrium sizes and high growth rates make the population persist longer and therefore favour evolutionary rescue (figures 2*b* and 3*c*). We also show in the electronic supplementary material that fixing an equal equilibrium size by adjusting the carrying capacity results in similar extinction times for all the growth types (see electronic supplementary material, figure S15). However, even at a fixed equilibrium size, the larger the growth rate, the larger the rescue probability (see electronic supplementary material, figure S15).

Many mathematical growth models have been developed to describe population demography, from the microscopic to the macroscopic scale, assuming different density-dependent growth [41]. Mathematical growth models allow for the fitting of population dynamics data to obtain, for example, growth rate estimates [69] and also allow for forecasting population dynamics [70]. However, to date, there is no universal model that best describes any dataset [42]. Our work highlights that it is crucial to correctly infer the growth type from empirical data when assessing the persistence of a population undergoing environmental change. The importance of density dependence depressing growth in assessing population persistence has already been emphasized in the field of population viability analysis [71], which investigates extinction risk and population growth or decline [72,73]. This aspect is missing in some theoretical studies that investigated evolutionary rescue assuming density-independent growth [74,75] and in some others that considered a ceiling-type carrying capacity limiting the population size but under which the growth is density-independent [31,76,77]. Although these assumptions about density dependence distinguish the contribution of evolutionary factors from ecological factors in evolutionary rescue mechanisms, they do not capture that many populations do not grow forever and have regulated growth. Similar to Chevin & Lande [78], we compared the impact of different density-dependent growths on evolutionary rescue. Although our model design is quite different from the one in [78], we also found that population regulation plays a critical role in the survival of the population. However, contrary to other models, our model decomposes the growth rate into a birth rate and a death rate that are distinct. As pointed out by Vinton & Vasseur [79], the environment likely applies different pressures on birth and death rates. Thus, combining birth and death rates into a single growth rate is an oversimplification that carries the risk of poorly assessing the survival of the population. The risk of misestimation arises because the same growth rate value, which can be obtained by different combinations of birth and death rate values, may result in different abilities to persist under environmental changes. This observation leads us to believe that an exciting extension of our work would be to investigate evolutionary rescue with environment-dependent death rates and compare them to our results with environment-dependent birth rates.

On the purely ecological side of our model, other possible extensions carry the potential for applications in conservation biology. For example, our extinction time calculation could allow for identifying harvesting strategies that avoid the extinction of exploited animal populations or crops [80,81]. In other words, the ecological part of our model could contribute to the problem of harvesting optimization, which has been investigated theoretically for a long time [82–84]. Harvesting optimization is critical to maintaining sustainable harvest and avoiding extinction due to over-exploitation [85]. Harvest models have shown that accounting for extinction risk and environmental fluctuations is crucial to choosing the harvest strategy that maximizes yield [83,84]. Similar to the model in [82], our model includes a growth rate and a death rate that can be interpreted as a harvest effort rate. Whereas many models focus on environmental fluctuations that continuously impact the growth rate [82], our model considers periods when animals reproduce and periods when they do not reproduce while dying in proportion to their numbers, which can be interpreted as proportional harvest. We show with our model that the persistence of the population is reduced if its reproduction periods are stochastic. It would be interesting to extend our model to a death rate that explicitly depends on the environment to model an irregular harvesting effort and assess its impact on population persistence and yield.

### (c) No significant differences in the impact of an imperfectly harsh environment on evolutionary rescue compared to a perfectly harsh environment

Our model compares the impact of a perfectly harsh environment (i.e. one that fully prevents births) to an imperfectly harsh environment (i.e. one that does not fully prevent births) on evolutionary rescue. We show no significant differences between the two harshness levels, especially for death rates much larger than birth rates. Specifically, we prove that the mean survival time in a harsh environment, and thus the mean total extinction time, is similar for both perfectly and imperfectly harsh environments (see electronic supplementary material, figure S6 and figure 2*c*). Although some births may occur in an imperfectly harsh environment, a mutant appearance is unlikely. Thus, an imperfectly harsh environment does not significantly favour evolutionary rescue compared with a perfectly harsh one (figure 3*d*).

This result means that our analytical results apply to an extensive range of scenarios where populations are exposed to an environment that successively causes their decline and growth. In particular, our analytical predictions for the perfectly harsh case are a good approximation for the case in which the environment does not fully prevent reproduction, which is likely to be the case in nature. In the case of a perfectly harsh environment, we emphasize that our analytical predictions are explicit and exact. They do not rely on a deterministic nor a diffusion approximation that has been shown to describe extreme events such as extinctions poorly [39], although widely used in population genetics [10].

### (d) Importance of de novo mutations in the presence of rapid environmental fluctuations

There are two main genetic routes for evolutionary rescue: a de novo mutation or a pre-existing mutation resulting from standing genetic variation. Our model allows for comparing of the two genetic routes and their contribution to the evolutionary rescue of a population caught in a fluctuating environment threatening its persistence. We show that de novo mutations are particularly important for environment durations shorter than the mean survival time of the population in a harsh environment. Specifically, de novo mutations allow the population to be rescued if the potential pre-existing mutation did not. Conversely, for environment durations longer than the mean survival time of the population in a harsh environment, de novo mutations do not have enough time to appear before population extinction. Because there are no de novo mutations, standing genetic variation is alone responsible for the evolutionary rescue, or not, of the population.

Orr & Unckless [31] investigated the evolutionary rescue of a population facing an abrupt environmental change threatening its survival unless a new or pre-existing mutant saves the population. They found that the initial proportion of mutants existing before the environmental change, the mutation rate and the wild-type fitness cost in the new environment define whether evolutionary rescue by new mutations is more likely than by pre-existing mutations. Our model, in which we consider periodic and quasi-periodic stress (or harsh) environments, allowed us to shed light on an additional criterium: environmental and survival time scales. Although our equation for the probability of rescue by de novo mutations has a similar form to the one derived in [86], our approach is more general as it does not assume time-discrete dynamics and, thus, applies easier to natural populations displaying continuous growth and overlapping generations [87]. Also, our approach includes density-dependent selection and growth, and applies to populations whose extinction is not deterministic since we calculate the mean extinction time in the absence of rescue (e.g. exponential decay after an abrupt change [31,86]). We hope that our model will pave the way to a new analysis of evolution data, as done in [87].

In summary, the randomness of environmental fluctuations is essential to consider when quantifying the persistence of a population, as is its growth type. Conversely, the harshness of the environment does not significantly impact the persistence of the population as long as it induces its decline.

## Data Availability

Simulations were performed with C (version gcc-9) and Matlab (version R2021a). All annotated code to repeat the simulations and visualizations is available on Zenodo (doi:10.5281/zenodo.7886833) [88]. Detailed simulation methods, additional results, and figures are provided in electronic supplementary material [89].
